# Healthcare Provider-Related Factors in the Diagnostic Delay of Cervical Cancer: A Cross-Sectional Study

**DOI:** 10.7759/cureus.61865

**Published:** 2024-06-07

**Authors:** Eljo P Sebi, Parvathi Tejanaik, Viswanath Narendiran

**Affiliations:** 1 Department of Obstetrics and Gynecology, Jawaharlal Institute of Postgraduate Medical Education and Research (JIPMER), Puducherry, IND; 2 Department of Preventive Medicine, Jawaharlal Institute of Postgraduate Medical Education and Research (JIPMER), Puducherry, IND

**Keywords:** pap smear, via, screening guidance, healthcare provider, advanced cervical cancer

## Abstract

Background and objective

Cervical cancer is the second most common malignancy among Indian women. In 2018, the World Health Organization (WHO) called for global action toward the elimination of cervical cancer through the triple-intervention strategy. One of its pillars is ensuring 70% screening coverage of eligible women with a high-performance test at least twice in their lifetime. Various factors contribute to the delayed diagnosis of cervical cancer, increasing the burden of the disease. In this study, we aimed to determine the healthcare provider (HCP)-related factors in the diagnostic delay of advanced cervical cancer.

Methods

This prospective cross-sectional study was conducted over two months in the cancer clinic of the Department of Obstetrics and Gynecology, Jawaharlal Institute of Postgraduate Medical Education and Research (JIPMER), Puducherry, India. We interviewed 384 women diagnosed with advanced cervical cancer [the International Federation of Gynecology and Obstetrics (FIGO) stage IB3-IVB] by using a questionnaire to capture data inputs regarding the various healthcare services they had received in the past 10 years along with details of HCPs. The collected data were analyzed using the software STATA version 17.0.

Results

Among 384 participants, 185 (48.1%) had interacted with an HCP in the past 10 years; 157 (40.8%) of them had visited a healthcare facility. Among these 185 women, only 22.16% had been advised to undergo screening, and only 15.18% had been tested despite several having access to primary health centers within 10 km of their residence. The lack of screening guidance by HCPs accounted for 78% of delayed diagnoses of cervical cancer.

Conclusions

Based on our findings, a deficiency in screening guidance in the asymptomatic period by healthcare providers across various levels of our healthcare system contributed significantly to the delayed diagnosis of cervical cancer.

## Introduction

Cervical cancer is the eighth most commonly diagnosed cancer in women worldwide, with an estimated 662,301 new cases and 348,874 deaths. As per GLOBOCON 2022, it ranks as the second most common malignancy among Indian women, with an estimated 1,27,526 new cases and 79,906 deaths [1]. In 2018, the World Health Organization (WHO) called for global action toward the elimination of cervical cancer (defined as ≤4 cases per 100,000 women worldwide), through the triple-intervention strategy. One of its pillars is ensuring 70% screening coverage of eligible women with a high-performance test at least twice in their lifetime [2]. 

Organized screening programs using the Papanicolaou test have dramatically reduced cervical cancer incidence and mortality in developed countries. Low-middle-income countries (LMICs) like India mainly rely on opportunistic screening. Visual inspection with acetic acid (VIA) has been an effective alternative screening method for the prevention of cervical cancer [3]. India has a multi-tiered system for cervical cancer screening, aiming to make it accessible for every woman. Primary health centers (PHCs) constitute the core medical system led by medical officers. They oversee various national programs at the village level with support from village health nurses (VHN), health assistants (both genders), and Accredited Social Health Activists (ASHA).

These health workers play a crucial role in communities, conducting home visits, maintaining family health records, and promoting preventive healthcare, including cervical cancer screening. With the launch of the operation framework of India for cancer screening in 2016, ASHA workers have been designated as key motivators for cervical cancer screening among women. It is recommended that all women aged 30-65 years undergo screening with VIA every five years at PHCs by medical officers (primary care physicians), nurses, and mid-level providers, including Auxiliary Nurse Midwives (ANMs). This approach ensures women can access screenings close to home by qualified personnel in well-equipped facilities while maintaining their privacy [4].

The delay in diagnosis in the preinvasive or early stage of cervical cancer is a multifaceted problem, attributed to various patient, clinical, sociodemographic, diagnostic, healthcare infrastructure, and therapeutic factors, contributing to advanced-stage disease. The patient-related factors mainly included lack of education, poor social background, social stigma, and cultural misconceptions, seeking treatment from traditional healers, discomfort with male providers, and fear of pain with internal examination. The healthcare provider (HCP)-related factors include a shortage of staff, lack of trained staff, and lack of knowledge of policy and cervical cancer screening guidelines. The systemic factors include low-quality healthcare infrastructure as well as insufficient space and supply issues for screening, causing diagnostic and therapeutic delays following the diagnosis of preinvasive and invasive lesions. Inadequate screening and delayed diagnosis are significant reasons behind women progressing to the advanced stage of cancer [5-12]. 

There is a lack of data regarding the HCP-related delays contributing to the screening of asymptomatic women in India. In this questionnaire-based cross-sectional study involving women diagnosed with advanced-stage cervical cancer, we aim to determine the HCP-related factors delaying the screening and diagnosing of preinvasive/invasive cervical lesions.

## Materials and methods

Study setting and participants

This prospective cross-sectional study was conducted in the cancer clinic of the Department of Obstetrics and Gynecology, Jawaharlal Institute of Postgraduate Medical Education and Research, Puducherry, India, over two months from November to December 2023. We diagnose and manage around 2500-2800 cervical cancer cases at our institute annually. The study protocol was approved by the Institutional Ethics Committee (JIP/IEC-OS/302/2023) before commencing the study, and we adhered to the provisions of the Declaration of Helsinki in conducting the study.

All newly diagnosed advanced cervical cancer cases [the International Federation of Gynecology and Obstetrics (FIGO) stage IB3-IVB] in the last six months and scheduled to undergo concurrent chemoradiation therapy (CCRT) or those currently on CCRT and incidentally diagnosed with invasive lesions following simple hysterectomy (abdominal/vaginal/minimal access) for benign gynecological conditions were included in the study after obtaining informed written consent. All women with vault cancer, local/ regional recurrence following radical surgery/CCRT for early-stage cervical cancer, and critically ill patients were excluded from the study.

Sample size calculation

Anticipating a 50% delay in cervical cancer screening/diagnosis attributed to HCP-related factors, with a 5% level of significance and 5% absolute precision, the sample size was estimated based on the single proportion formula to be 384. A convenient sampling technique was employed.

Data collection

Data was collected using a pre-designed case record form and questionnaire to capture the data inputs regarding the various healthcare services received in the past 10 years along with the details of HCPs. The questions were asked in patients' vernacular language. The clinical details and biopsy reports were retrieved from medical records.

Statistical analysis

The collected data were entered into an MS Excel sheet and analyzed using Stata Statistical Software: Release 17 (StataCorp LLC, College Station, TX). A histogram with a normal curve and Q-Q plot was used to assess the normality through the eyeballing technique. Continuous variables such as age and duration of comorbidity were expressed as mean with standard deviation (SD) or median with interquartile range (IQR) depending on the data distribution. Categorical variables were expressed as frequencies with percentages. The Chi-square test was used to compare two categorical variables. A p-value less than 0.05 was considered statistically significant.

## Results

Sociodemographic and clinical characteristics of the study participants

A total of 384 participants who fulfilled the inclusion and exclusion criteria were included in the study. The sociodemographic and clinical characteristics of study participants are presented in Table 1.

**Table 1:** Sociodemographic and clinical characteristics of study participants (N=384) ^a^Modified BG Prasad Classification 2023 FIGO: the International Federation of Gynecology and Obstetrics

Characteristic	Frequency	Percentage
Age, years		
≤40	20	5.21
>40	364	94.79
Socioeconomic status^a^		
Lower class	268	69.79
Lower middle class	86	22.40
Middle class	24	6.25
Upper middle class	6	1.56
Residence		
Urban	46	11.98
Rural	338	88.02
Distance to nearby health facility, km		
≤10	346	90.10
>10	38	9.90
Type of nearest healthcare facility		
Government	348	90.62
Private	36	9.38
Educational status		
Illiterate	214	55.73
Lower primary	88	22.92
Upper primary	44	11.46
High school	32	8.33
Higher secondary	6	1.56
Religion		
Hindu	373	97.14
Christian	5	1.30
Muslim	6	1.56
Marital status		
Married	280	72.92
Widow	103	26.82
Divorced	1	0.26
Parity status		
Nulliparous	4	1.04
Primiparous	18	4.69
Multiparous	362	94.27
Menopausal status		
Premenopausal	62	16.15
Postmenopausal	322	83.85
Comorbidities	118	30.73
Hypertension	25	6.51
Diabetes mellitus	41	10.68
Thyroid disorders	6	1.56
Arthritis	1	0.26
Other cancer	1	0.26
>1 comorbid condition	44	11.46
Symptoms		
White discharge per vagina	184	47.92
Foul-smelling discharge	20	5.21
Post-coital bleeding	29	7.55
Intermenstrual bleeding	25	6.51
Postmenopausal bleeding	91	23.70
Lower abdominal pain	29	7.55
Low backache	6	1.56
Duration of symptoms, years		
<1	246	64.1
1-5	133	34.6
>5	5	1.3
Histology		
Squamous cell carcinoma	343	89.3
Adenocarcinoma	41	10.7
FIGO stage at diagnosis		
1B3	13	3.38
IIA1-IIB	174	45.31
IIIA-IIIC2	181	47.14
IV	16	4.17

The mean age of participants was 56.24 ± 10.5 years (range: 27-87 years). More than half of the participants were illiterate (55.7%), followed by those with a lower primary education (22.9%). About two-thirds of the participants belonged to the lower socioeconomic class (69.7%). Three-fourths of the participants resided in rural areas (88%), with the nearest health facility (primary health center) within 10 km of their residence. Most women were homemakers (87.24%). Nearly three-fourths of the participants were married (72.9%), and one-third were widows. Of note, 94.27% were multiparous, and 83.85% were postmenopausal at the time of presentation to the healthcare facility.

One-third of the study participants suffered from other non-communicable conditions (30.7%), with a mean duration of 6.34 ± 3.7 years. More than one-third of them suffered from more than one comorbid condition, the most common being type 2 diabetes mellitus (DM), followed by systemic hypertension and thyroid disorders; 97% were on three-monthly follow-ups with their HCP (medical officer/physician).

More than half of the study participants' trigger symptoms for consultation were white discharge and foul-smelling vaginal discharge, followed by postmenopausal bleeding. Two-thirds of the women had more than one clinical symptom. Most of them had symptoms for less than a year. Nearly 90% of cervical cancers were of squamous cell carcinoma (SCC) pathology. The majority of them were in stage II (45.31%) and III (47.14%) at the time of initial consultation; hence, they were scheduled for or were already undergoing CCRT. Less than 5% of them were diagnosed with stage IV and were on palliative radiotherapy or chemotherapy.

Delay in cervical cancer screening due to healthcare provider-related factors

Among the 384 participants interviewed, 185 (48.18%) participants had engaged with an HCP in the past 10 years; 157 (40.89%) of them had visited a healthcare facility. These visits did not involve consultations for acute illnesses such as viral fever and acute gastroenteritis. Of note, 82 (44.3%) participants had consulted a medical officer, 51 (27.9%) had visited a physician, 32 (16.4%) a VHN/ANM/ASHA worker, and 20 (10.9%) had a consultation with a gynecologist. These consultations included visits to their nearby primary health center (54.1%), followed by health camps (15.3%), community health centers (9.6%), district hospitals (9.6%), private health facilities (6.3%), and medical colleges (5.1%).

**Figure 1:**
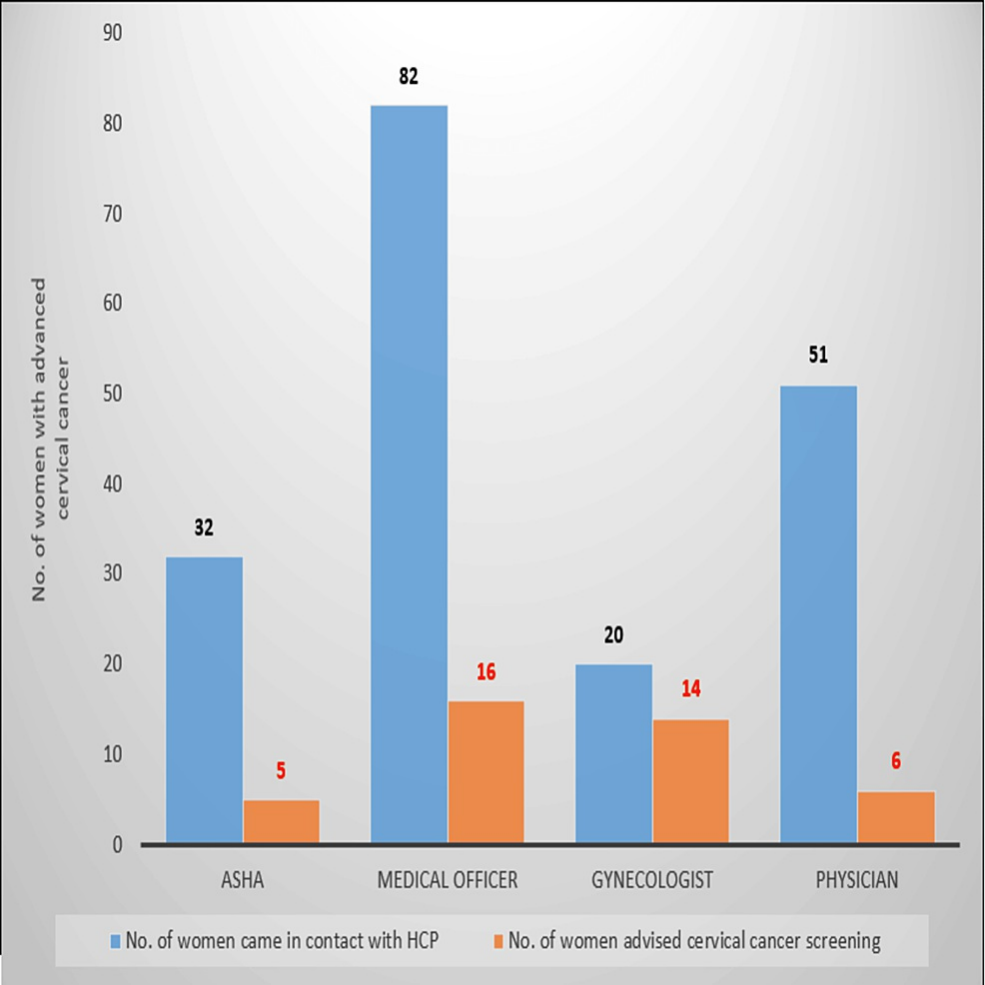
Women who received cervical cancer screening advice among those who contacted an HCP during their asymptomatic period ASHA: Accredited Social Health Activists; HCP: healthcare provider

Overall, 10.68% of the study participants were advised to undergo cervical cancer screening in the asymptomatic period in the past 10 years. Among the participants who interacted with HCPs, only 41 (22.16%) were advised to undergo cervical cancer screening (VIA/VILI/Pap smear), and 29 (15.68%) underwent internal examination and screening tests in the last decade. The proportion of screening advice provided by various HCPs is shown in Figure 1. It was found that 14 (70%) of the women who consulted with a gynecologist were advised to undergo screening. In contrast, only five (15.6%), 16 (19.5%), and six (11.7%) participants who consulted with ASHA workers, medical officers, or physicians were advised to undergo screening, respectively.

There was no association between the participants’ educational level and distance from the health facility with cervical cancer screening.

## Discussion

Cervical cancer is the second most common cancer among Indian women. In this study, our objective was to assess the HCP-related factors contributing to the delayed diagnosis of cervical cancer. We assessed the sociodemographic, other non-communicable comorbidities, clinical history, and HCP-related factors among recently diagnosed cases of advanced cervical cancer. In our study, most women were illiterate and belonged to rural regions, but had access to a primary health center within 10 km of their residence. Access to healthcare facilities is one of the major determinants of early diagnosis and treatment of cancer. The infrastructure established for primary healthcare (PHCs and CHCs) caters to approximately 30,000 populations for a PHC and one CHC for a block in India. This brings primary healthcare near to the residences of individuals. There was no association between the education of the participants and distance from the healthcare facility and their getting screening advice for cervical carcinoma screening from HCP, which aligns with the findings of a study done in Botswana [5].

Most of the women in our study presented to healthcare facilities with symptoms of less than a year's duration, and they presented with abnormal vaginal discharge and postmenopausal bleeding, similar to other studies [4,6]. One-third of the women had coexisting medical conditions, the most common being type 2 DM, requiring frequent follow-up with their HCPs, similar to the study by Bhatia et al. [5]. A hierarchical model analysis of risk factors for cervical cancer in two Brazilian hospitals showed that 40% of the women with invasive cervical lesions had coexisting chronic diseases, and the majority of them were under regular follow-up [13]. In countries with opportunistic screening for cervical cancer, these visits to their HCPs provided the only opportunity for screening advice or testing, which was missed in most of the women, leading to delayed diagnoses.

While the previous studies in the literature have assessed the delays in diagnosis at various levels, such as patient, referral, diagnostic, and HCP, there is a lack of standardized definition of these delays [4-11,14]. HCP-related delays have been assessed mainly in terms of their training and knowledge of guidelines and policies of cervical cancer screening programs [7-10]. In our study, we assessed the HCP delays in terms of cervical cancer screening advice received by women diagnosed with advanced cervical cancer in the past 10 years from various HCPs with whom they had consulted.

We had assumed that HCP factors contributing to delayed diagnosis would account for 50% of advanced cervical cancer cases. Our study results showed that about 78% of the women had not received any advice or screening for cervical cancer following contact with an HCP in the past decade. The study by Lim et al. [8] reported a 60% provider-related delay in terms of not visualizing the cervix in symptomatic women. Similar delays have been noted during audits of general practices in London regarding other malignancies, where about one-quarter of the patients had three or more visits to HCPs before diagnosis of cancer and referral [15]. We noted that despite the majority of the women consulting with a medical officer, physician, and VHN/ASHA worker, they failed to receive advice for cervical cancer screening. Two-thirds of the women consulting with a gynecologist received screening. There is limited data in the literature comparing cervical cancer screening concerning various HCPs.

The major strength of our study is that we assessed the screening advice received in the past decade in women with advanced cervical cancer from HCPs at all levels. However, the study has a few limitations, such as the potential for recall bias. Also, we were unable to capture data related to the training and knowledge of HCPs and the screening service delivery barriers at the healthcare facilities. These factors can further be studied at the level of each healthcare facility in our country by involving the HCPs of various disciplines.

Clinicians in low-middle-income countries like India mainly rely on opportunistic screening for cervical cancer. With increasing incidences of other non-communicable diseases like hypertension and DM requiring frequent visits to HCPs, it becomes more crucial to integrate the screening program with other existing health programs and involve medical specialists from other disciplines to educate and sensitize women on cervical cancer screening for broader coverage. We sincerely believe that our findings would aid policymakers audit ongoing national screening programs and strengthen the strategies for opportunistic screening methods to increase the screening coverage of eligible women. It would guide the implementation of structured training programs for HCPs at all levels and specializations to meet the WHO target of global elimination of cervical cancer by 2030.

## Conclusions

This study highlights the lack of screening advice by HCPs at different levels of our healthcare system, causing delayed diagnoses of cervical cancer. The screening program for early cervical cancer detection should be available at the level of all HCPs to achieve 70% screening coverage among the eligible target population. To achieve the goal of the global elimination of cervical cancer by 2030, we need the integration of various other national health programs with cervical cancer screening for wider coverage. Also, there is an urgent need to sensitize various stakeholders about the disease burden and involve other healthcare disciplines to achieve the goal.

## References

[1] International Agency for Research on Cancer (2024). International Agency for Research on Cancer: cervical cancer incidence and mortality 2022. https://gco.iarc.who.int/media/globocan/factsheets/cancers/23-cervix-uteri-fact-sheet.pdf.

[2] (2024). WHO guideline for screening and treatment of cervical pre-cancer lesions for cervical cancer prevention, second edition. https://www.who.int/publications/i/item/9789240030824.

[3] Sankaranarayanan R, Esmy PO, Rajkumar R (2007). Effect of visual screening on cervical cancer incidence and mortality in Tamil Nadu, India: a cluster-randomised trial. Lancet.

[4] (2024). Ministry of Health and Family Welfare Government of India: operational framework: management of common cancer. https://main.mohfw.gov.in/sites/default/files/Operational%20Framework%20Management%20of%20Common%20Cancers_1.pdf?utm_medium=email&utm_source=transaction.

[5] Friebel-Klingner TM, Luckett R, Bazzett-Matabele L (2021). Clinical and sociodemographic factors associated with late stage cervical cancer diagnosis in Botswana. BMC Womens Health.

[6] Bhatia RK, Rayne S, Rate W (2018). Patient factors associated with delays in obtaining cancer care in Botswana. J Glob Oncol.

[7] Dereje N, Addissie A, Worku A (2020). Extent and predictors of delays in diagnosis of cervical cancer in Addis Ababa, Ethiopia: a population-based prospective study. JCO Glob Oncol.

[8] Lim AW, Ramirez AJ, Hamilton W, Sasieni P, Patnick J, Forbes LJ (2014). Delays in diagnosis of young females with symptomatic cervical cancer in England: an interview-based study. Br J Gen Pract.

[9] Rosser JI, Hamisi S, Njoroge B, Huchko MJ (2015). Barriers to cervical cancer screening in rural Kenya: perspectives from a provider survey. J Community Health.

[10] Maseko FC, Chirwa ML, Muula AS (2015). Health systems challenges in cervical cancer prevention program in Malawi. Glob Health Action.

[11] Nkurunziza C, Ghebre R, Magriples U, Ntasumbumuyange D, Bazzett-Matabele L (2021). Healthcare provider challenges to early detection of cervical cancer at primary healthcare level in Rwanda. Gynecol Oncol Rep.

[12] Gyenwali D, Khanal G, Paudel R, Amatya A, Pariyar J, Onta SR (2014). Estimates of delays in diagnosis of cervical cancer in Nepal. BMC Womens Health.

[13] Lourenço AV, Fregnani CM, Silva PC, Latorre MR, Fregnani JH (2012). Why are women with cervical cancer not being diagnosed in preinvasive phase? An analysis of risk factors using a hierarchical model. Int J Gynecol Cancer.

[14] Mumba JM, Kasonka L, Owiti OB (2021). Cervical cancer diagnosis and treatment delays in the developing world: Evidence from a hospital-based study in Zambia. Gynecol Oncol Rep.

[15] Mayor S (2011). A quarter of patients with cancer see their GP several times before being referred. BMJ.

